# Infrared optical properties modulation of VO_2_ thin film fabricated by ultrafast pulsed laser deposition for thermochromic smart window applications

**DOI:** 10.1038/s41598-022-15439-5

**Published:** 2022-07-06

**Authors:** Eric Kumi Barimah, Artitsupa Boontan, David P. Steenson, Gin Jose

**Affiliations:** 1 School of Chemical and Process Engineering, University of Leeds, Clarendon Road, Leeds, LS2 9JT UK; 2 School of Electronic and Electrical Engineering, University of Leeds, Clarendon Road, Leeds, LS2 9JT UK

**Keywords:** Energy science and technology, Engineering, Materials science, Nanoscience and technology, Optics and photonics

## Abstract

Over the years, vanadium dioxide, (VO_2_(M1)), has been extensively utilised to fabricate thermochromic thin films with the focus on using external stimuli, such as heat, to modulate the visible through near-infrared transmittance for energy efficiency of buildings and indoor comfort. It is thus valuable to extend the study of thermochromic materials into the mid-infrared (MIR) wavelengths for applications such as smart radiative devices. On top of this, there are numerous challenges with synthesising pure VO_2_ (M1) thin films, as most fabrication techniques require the post-annealing of a deposited thin film to convert amorphous VO_2_ into a crystalline phase. Here, we present a direct method to fabricate thicker VO_2_(M1) thin films onto hot silica substrates (at substrate temperatures of 400 °C and 700 °C) from vanadium pentoxide (V_2_O_5_) precursor material. A high repetition rate (10 kHz) femtosecond laser is used to deposit the V_2_O_5_ leading to the formation of VO_2_ (M1) without any post-annealing steps. Surface morphology, structural properties, and UV–visible optical properties, including optical band gap and complex refractive index, as a function of the substrate temperature, were studied and reported below. The transmission electron microscopic (TEM) and X-ray diffraction studies confirm that VO_2_ (M1) thin films deposited at 700 °C are dominated by a highly texturized polycrystalline monoclinic crystalline structure. The thermochromic characteristics in the mid-infrared (MIR) at a wavelength range of 2.5–5.0 μm are presented using temperature-dependent transmittance measurements. The first-order phase transition from metal-to-semiconductor and the hysteresis bandwidth of the transition were confirmed to be 64.4 °C and 12.6 °C respectively, for a sample fabricated at 700 °C. Thermo-optical emissivity properties indicate that these VO_2_ (M1) thin films fabricated with femtosecond laser deposition have strong potential for both radiative thermal management or control via active energy-saving windows for buildings, and satellites and spacecraft.

## Introduction

Increasingly vanadium dioxide (VO_2_) (M1) is a technologically important metal oxide, owing to its remarkable change in first-order insulator-to-metal transition (IMT) at a critical temperature of around 68 °C^1,2^. The phase transition temperature of VO_2_ (M1) thin films can be triggered using external stimuli such as thermal, electrical, and ultrafast optical excitations. The induced phase transition of VO_2_ thin films from monoclinic insulator to rutile metallic phase is reversible and accompanied by a large change in electrical, magnetic and optical properties. These characteristics have significant potential for a wide range of modern applications such as actuators, passive smart radiation devices, thermochromic smart (active) windows, modulation of near-infrared (NIR) to mid-infrared (MIR) wavelengths or optical switching to modulate the MIR emissivity, and passive radiative cooling^3–20^. For instance, the phase transition temperature of VO_2_ (M1) thin film is associated with modulating NIR to MIR spectral range transmittance and reflectance as a function of temperature. These properties could be utilised to develop more efficient thermal control systems^2,14^ depending on the IR substrate on which VO_2_ film is deposited and its thickness. Changes in optical properties of VO_2_ (M1) thin film in the MIR is quite useful for specific applications including spacecraft thermal control, energy-saving buildings and selective camouflage against IR sensors.

There had been numerous studies on VO_2_ (M1) thin films (thickness < 0.90 μm) for visible and near-infrared (NIR) thermochromic energy-saving applications^19–23^. Such VO_2_ films exhibit outstanding NIR (1.0 to 2.5 μm wavelength) transparency (> 70% transmittance) at low temperatures of about 25 °C. However, the transmittance is completely blocked or reduced to nearly zero at temperatures above the 68 °C metal-to-insulator transition. These studies demonstrate better control of the insulator–metal transition switching properties in the NIR wavelength range, but there are limited numbers of comparative studies on VO_2_ (M1) films operating in the MIR-to-longer wavelength region (LWIR). Guinneton et al.^15^ in 2001, fabricated VO_2_ thin films on silica substrates in thicknesses less than 200 nm using a vanadium target and RF reactive sputtering to evaluate controllable optical properties in the infrared. Similarly, Gianmario et al.^16^ deposited VO_2_ thin films on a silicon wafer using the same RF sputtering methods to estimate the optical properties and thermal hysteresis in the MIR sub-spectral ranges. Naturally, both examples required a post-deposition annealing stage. A transition temperature around 68 °C was reported with a significant difference in the thermal hysteresis bandwidth at the short and long-wavelength regions. Recently, Dongqing et al.^23^ synthesised VO_2_ thin films of thicknesses 400 nm and 900 nm using a sol–gel process to evaluate thermochromic phase transitions and IR thermochromic property’s in the 7.5–14 μm wavelength range.

Over the past few decades, VO_2_ (M1) nanostructure thin films have been fabricated by using various methods, which include sol–gel, chemical vapour deposition, sputtering, atomic layer deposition, and nanosecond(ns) or femtosecond (fs) pulsed laser deposition (PLD)^18^. However, the majority of these deposition techniques are limited to the synthesis of VO_2_ films of less than 400 nm and require essential post-annealing processing to convert the various amorphous VOx phases to crystalline VO_2_ (M1). Consequently, there is a need to develop a suitable method capable of synthesising thicker VO_2_ (M1) films and ideally without post-annealing. As a consequence, fs-PLD offers the exceptional advantage of producing nanostructures of different particle sizes/thin film thicknesses, morphology, and chemical composition by fine-tuning the laser parameters (laser energy, repetition rate, and pulse width) and chamber conditions (gas pressure, substrate temperature, and substrate-target distance) in a single-deposition process. Conceivably this can also be done at speed and large scale. For example, the ablation mechanism of the fs-PLD is completely different from that of ns-laser PLD with an average ablation rate around 35 times higher than conventional ns-PLD; reported elsewhere^19^. We have recently demonstrated a sharp and abrupt metal-to-insulator transition (MIT) of three-to-four orders of magnitude resistivity change in thicker high-quality VO_2_(M1) films on sapphire substrates using the fs-PLD with a laser of repetition rate 10 kHz^1^. To the best of our knowledge, there has not been any report on fs-PLD with a repetition rate higher than this for fabricating of VO_2_ thin film onto silica substrate, and the significance of which is high deposition rates of high-quality materials.

In this study, we investigated the optimum conditions for the synthesis of thick VO_2_ (M1) films on silica substrate using a high repetition rate (10.0 kHz) fs-PLD technique. Significant parameters, including substrate temperature, surface morphology, optical band gap and refractive index in the UV–vis-NIR spectrum, and transition-switching in the MIR are discussed and reflect the potential application range of such materials.

## Experimental details

### Sample fabrication

Two VO_2_ (M1) thin films were fabricated onto silica substrates using a vanadium pentoxide (V_2_O_5_) target as reported previously by Kumi-Barimah et al.^1^. The silica substrates of sizes 20 mm × 30 mm × 1 mm were cleaned in an ultrasonic bath using acetone, followed by an isopropyl alcohol rinse then dried with clean lens tissue. The substrate and the target were mounted to respective holders in the PLD chamber, which was pumped down to a base pressure of 10^–7^ Torr prior to the process run, and then injected with high-purity process oxygen to a pressure of 70 mTorr. The substrate temperature was held at 400 °C (Sample code VT400) and 700 °C (Sample code VT700) with a substrate-to-target distance of 70 mm, for both. The deposition process used a laser fluence of 0.27 J/cm^2^ to ablate the V_2_O_5_ target for a period of 2 h using KMLabs Wyvern™ 1000–10 solid-state Ti:sapphire laser/amplifier. Samples VT400 and VT700 have growth rates of 6.25 nm/s and 5.42 nm/s with thin-film thicknesses of ~ 750 nm and ~ 650 as the deposition rate depends mainly on laser fluence and substrate temperature.

### Characterisation

The surface morphology and cross-sections of the VO_2_ (M1) thin films were prepared and characterized using a high-resolution monochromated field emission scanning electron microscope (FEG-SEM) with precise, focused ion beam (FIB) (FEI Helios G4 CX DualBeam). Furthermore, the VO_2_ (M1) thin films were analysed for elemental identification based on cross-sectional compositional contrast of the different atomic numbers via high-resolution transmission electron microscopy (HRTEM) and scanning (S)/TEM EDX spectroscopy imaging (FEI Tecnai F20 200 kV FEGTEM). Additionally, X-ray diffraction (XRD) pattern analysis of the prepared thin films was done using a Philips PANalytical X'pert Diffractometer, using Cu Kα radiation (λ = 1.54056 Å), at 40 kV and 100 mA. Each scan was performed with a diffractometer angle varied between 5° and 80° in a step size of 0.033°. A Perkin Elmer UV/VIS/NIR Lambda 950 spectrometer was also used to gather the transmittance and reflectance spectra at room temperature from 250 to 2500 nm to determine the optical band gap and complex refractive index of the samples under test. Furthermore, the MIR and LWIR (2500 nm to 25,000 nm) optical transmittance and reflectance were measured by Bruker Vertex 70v transmittance FTIR spectrometer, together with an A513/Q variable angle reflection accessory. The VO_2_ thin films were mounted on a heated stage to vary the sample temperature from 25 to 100 °C in the step of 10 °C increment during the study. The samples were allowed to reach a steady temperature during the heating stage before MIR transmittance was recorded to determine the thermochromic transition temperature and hysteresis width. The reflectance measurement was done using a variable angle reflection accessory (A513/Q Vertex 70v, Bruker) at an incidence angle of 20° and film temperatures of 25 °C, 60 °C, and 100 °C to determine the MIR emissivity.

## Results and discussion

### Surface morphological and structural evolution

**Figure 1:**
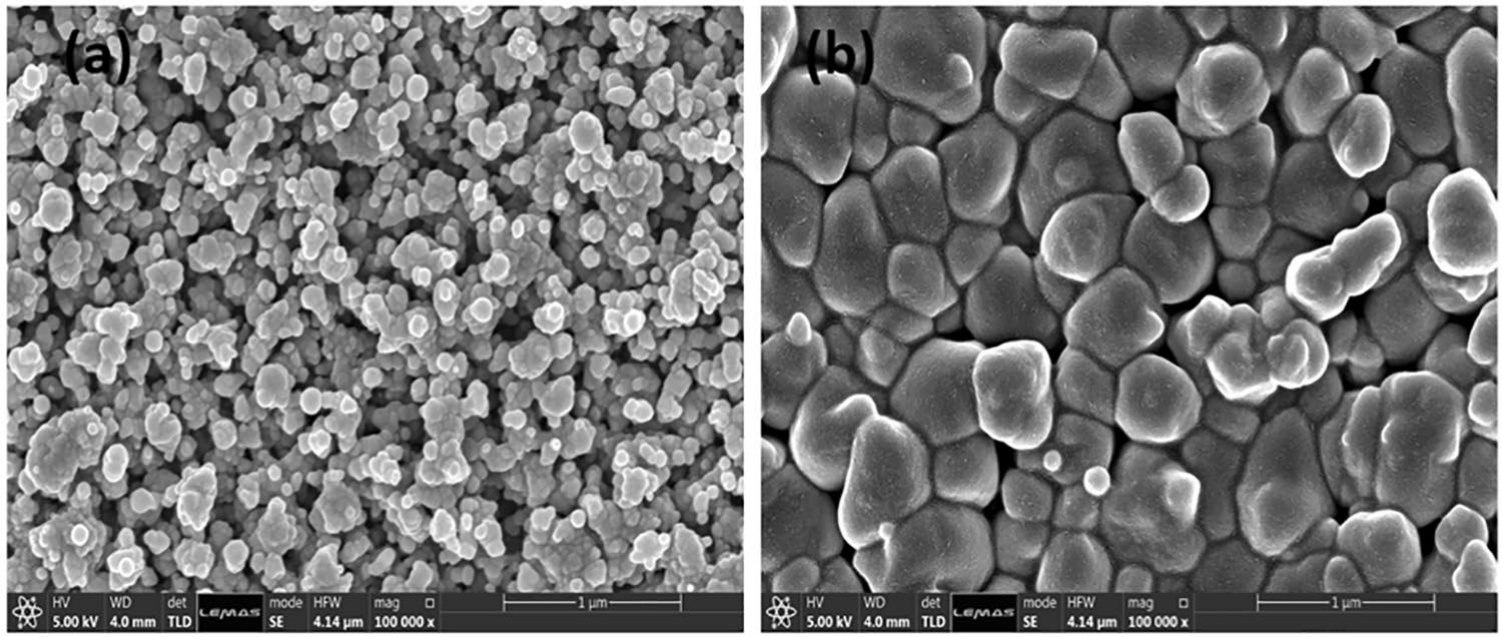
Top-view SEM images of the VO_2_ thin film deposited: VT400 [(**a**)], and VT700 [(**b**)].

The surface morphology of VO_2_ thin film samples fabricated was initially characterised by SEM imaging to evaluate substrate temperature effect on VO_2_ particles or grain size when deposited onto the silica substrate. Figure 1a,b show the top-view SEM images of the samples prepared at the substrate temperature of 400 °C and 700 °C. Sample VT400 °C exhibits a more uniform particle size distribution and pores with an average grain size of about 12 nm (according to ImageJ software analysis). On the other hand, adjusting deposition temperature to 700 °C larger and denser particles with an average grain size of 460 nm was obtained. Sample VT400 on the other hand consists of a particulate film which is a more coarse, loose, and porous structure.

**Figure 2:**
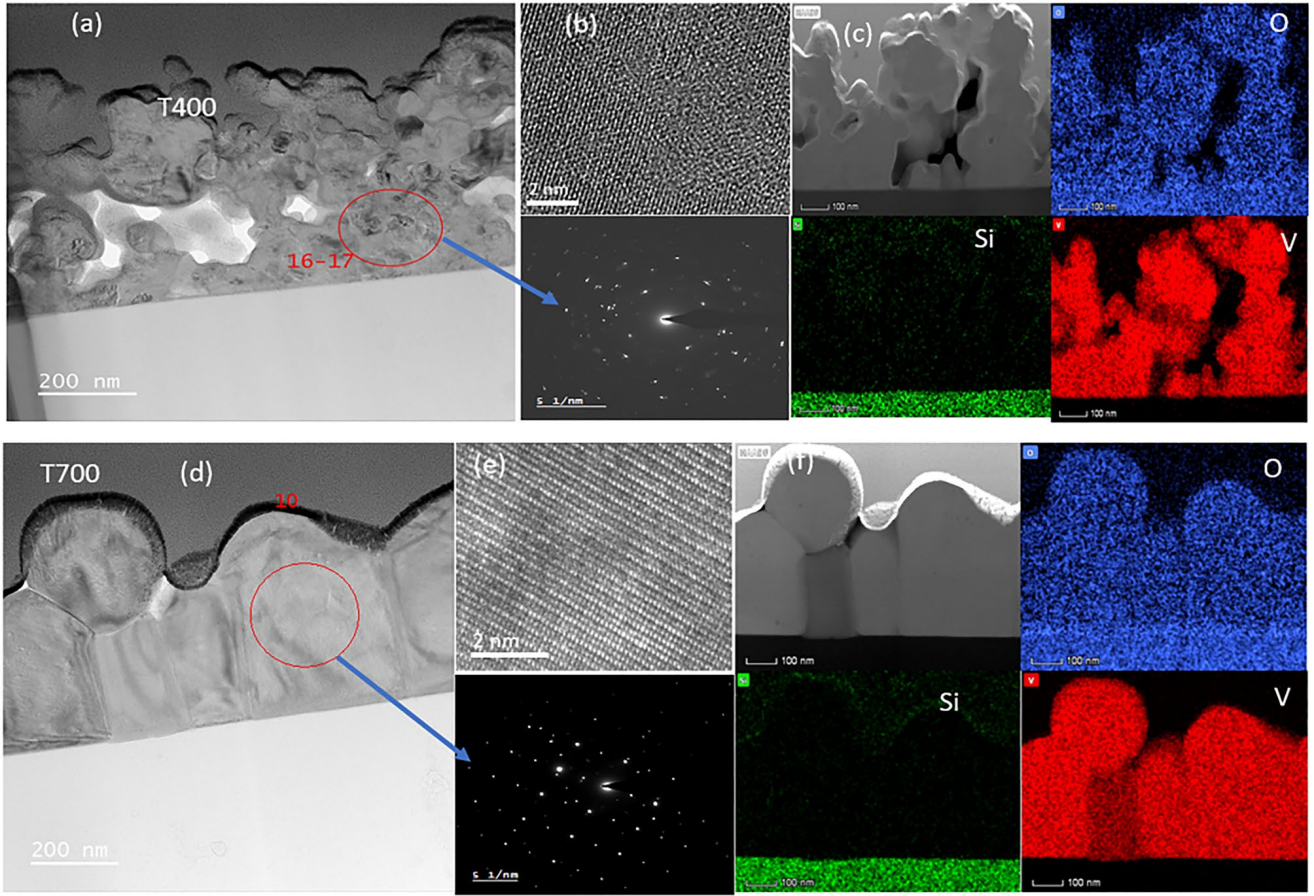
TEM cross-section image of VO_2_ thin films deposited onto silica substrates at temperatures of (**a**) 400 °C (VT400) and (**b**) 700 °C (VT700); (**b**,**e**) corresponding HRTEM and SAED patterns; (**c**,**f**) EDS chemical mapping obtained during cross-sectional HAADF-STEM analysis of different types of atoms(V, O, and Si).

The TEM cross-section of the samples VT400 and VT700 was prepared by focussed ion beam etching and mounting as illustrated in Fig. 2a,d, respectively. These lamellae were cut and mounted on TEM stubs for analysis and had average lamellae thicknesses of ~ 750 nm and ~ 650 nm with growth rates of 6.25 nm/s and 5.42 nm/s. The TEM cross-section of sample VT700 (2d) evidences the homogenous metastable state of VO_2_ film as compared to sample VT400 (2a), which is more porous (overly bright and dark areas in the image). These clearly show that the higher deposition temperature contributes to the nucleation and amalgamation of the denser polycrystalline material. Furthermore, the crystallinity of the samples was examined at the atomic scale by means of using a high angular dark field (HAADF) STEM image and selected area electron diffraction (SAED) pattern. Figure 2b,e illustrate HAADF-STEM and SAED patterns of samples VT400 and VT700 with VT400 exhibiting polycrystalline structure due to existing short-range order. On the other hand, the SAED pattern of sample VT700 confirms extended long-range polycrystalline structure owing to a discrete spot with a high degree of periodic order in the crystal lattice.

**Figure 3:**
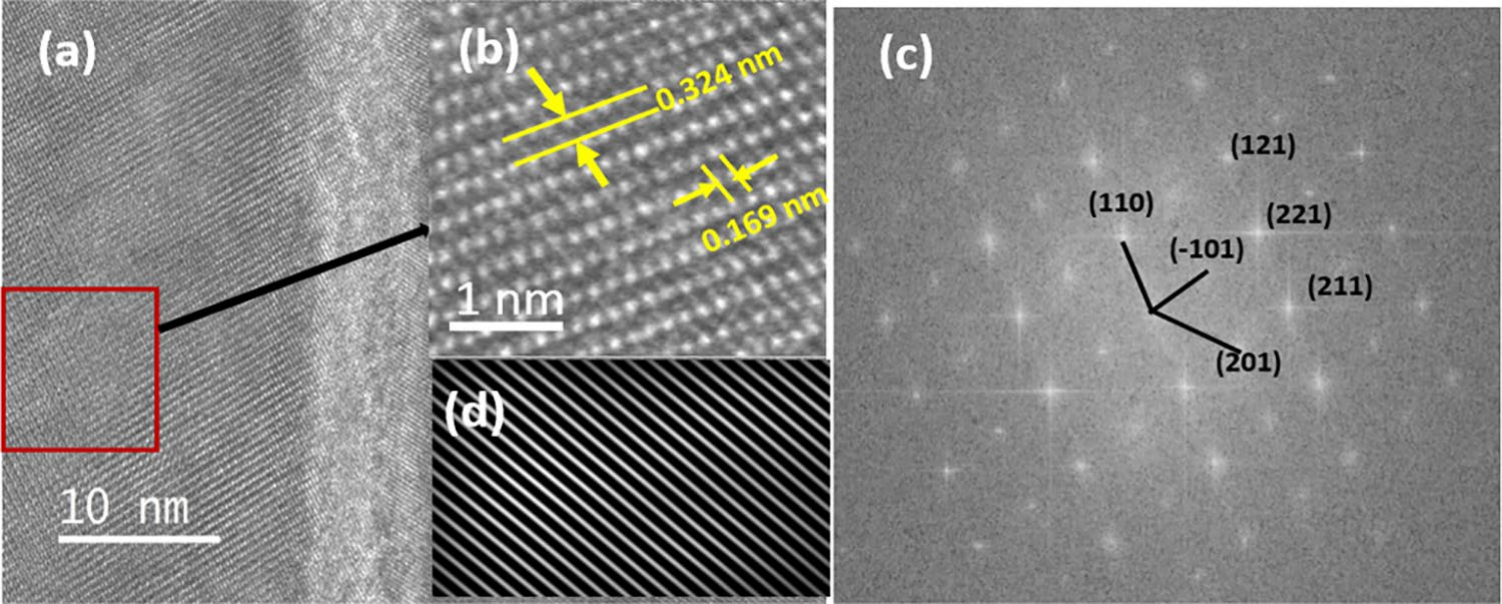
(**a**) HRTEM cross-sectional image of sample VT700; (**b**) Magnified HRTEM image of the rectangular marked region; (**c**) diffraction pattern of the crystallites of VO_2_; (**d**) inverse FFT Lattice fringe obtained from out-of-plane spacing of 0.324 nm.

To more quantitatively evaluate the crystallographic properties we carried out a Fast Fourier Transform (FFT) analysis to determine the d-spacing of the HAADF-STEM images. Figure 3a illustrates the HAADF-STEM cross-sectional image obtained from sample VT700 for crystallographic orientation evaluation. The HRTEM image extracted from the red rectangular region in Fig. 3a inset as Fig. 3b was employed to envisage the diffraction pattern and d-spacing depicted in Fig. 3c. The interplanar spacing attained matching to a d-spacing or an out-of-plane spacing of 0.324 nm, which correlates with the (110) plane of the VO_2_ (M1) phase. Similarly, in-plane spacing was found to be 0.169 nm corresponding to (221) plane with its lattice fringe shown in Fig. 3d. The interplanar spacing obtained from the FFT analyses and SAED pattern matches with a monoclinic structure of VO_2_ (M1). In addition, the diffraction quality of sample VT400 was assessed by examining the lattice crystal with the FFT of the image and comparing it with the SAED pattern (Fig. 2b) obtained from the HRTEM image. This reveals an interplanar spacing of 0.328, 0.245, 0.219, 0.169, and 0.146 nm, which correspond to lattice parameters of the (110), (011), (− 111), (221), and (213), respectively.

Additionally, we also analysed the elemental composition of samples VT400 and VT700 by using HAADF-STEM cross-sectional images. The STEM-EDX of these samples confirms a uniform distribution of elemental species such as vanadium (V) and oxygen (O) on the deposited layer without any intermixing between VO_2_ layer and silica substrate as depicted in Fig. 2c,f.

**Figure 4:**
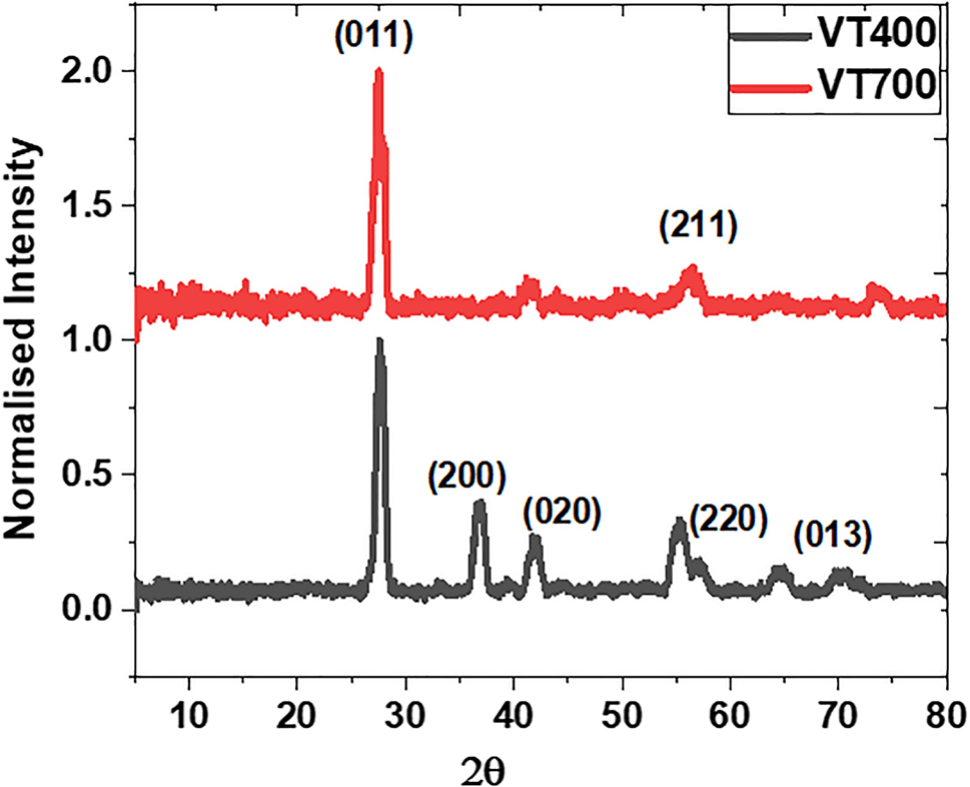
XRD diffraction patterns spectra of the VO_2_ thin films fabricated at different substrate temperatures 400 °C (VT400) and 700 °C (VT700).

Following the precise FIB, TEM and FFT examination of the thin film samples, XRD was performed to measure the crystallographic structure of the VO_2_ (M1) thin films deposited on silica substrates. Figure 4 illustrates the XRD pattern obtained from the samples VT400 and VT700 as prepared. Sample VT400 reveals six crystalline peaks centred at 2θ = ∼27.5°, ∼37.1°, ∼42.2°, ∼56.9°, ∼65°, and ∼73.5°, which correlate to (011), (200), (210), (220), (013), and (231). This confirms that sample VT400 is a polycrystalline material in good agreement with the SAED pattern observed from the HRTEM analysis. Moreover, as the substrate temperature was increased to 700 °C the XRD pattern exhibited one intense peak occurred at 2θ = 27.95° and a minor orientation peak at 56.9°. These peaks match with XRD patterns of (011) and (220) indicating a highly texturized polycrystalline VO_2_(M) structure and also correlated with FFT analysis shown in Fig. 3c. The XRD diffraction patterns correlate with the ICCD card numbers: 00-052-0794, 01-083-8516, and 04-007-1362 of a monoclinic (M1) crystal structure and P21/c phase group.

### Vis–NIR optical properties

**Figure 5:**
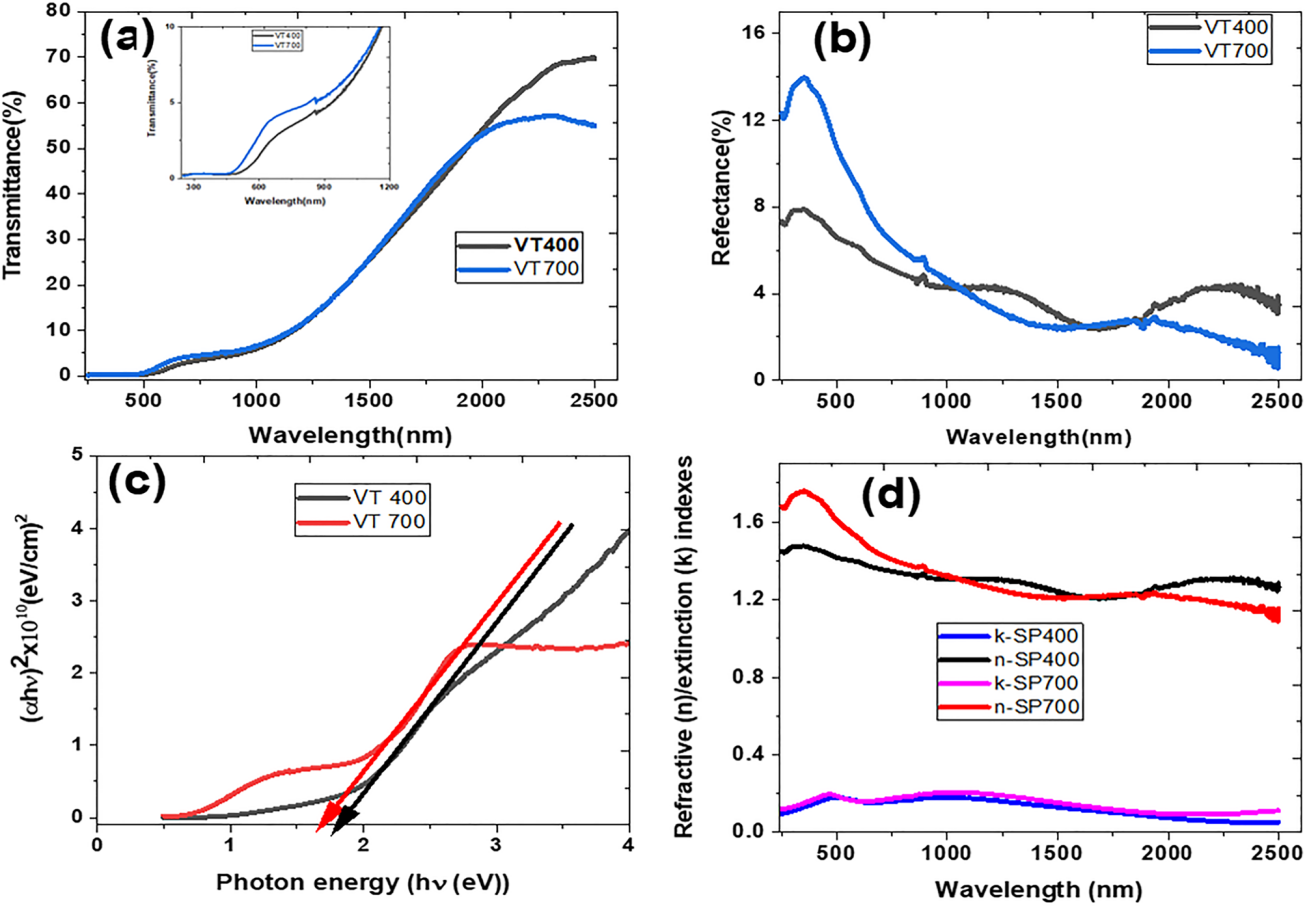
NIR optical properties of VO_2_ thin films deposited on silica substrate at different temperatures of 400 and 700 °C; (**a**) Transmittance, (**b**) reflectance, (**c**) optical band gap for $n = 1/2$, and refractive indices.

The optical transmittance and reflectance spectra of the VO_2_ thin films were measured by UV–VIS–NIR Spectrophotometer (PerkinElmer, LAMBDA 950) equipped with a 60 mm integrating sphere module in the spectral range of 250–2500 nm wavelength; which are presented in Fig. 5a,b. As shown in Fig. 5a, the transmittance for both samples fabricated at different substrate temperatures remained the same for wavelengths from 250 to 500 nm; however, the absorption edge which is sensitive to the thin film fabrication substrate temperature increases from 500 to 1200 nm [shows in the inset of Fig. 5a]. The absorption edge for samples VT400 and VT700 occurred at ~ 503 nm and ~ 470 nm. On the other hand, the transmittance decreased slightly with increasing substrate temperature in the NIR spectra range, which could be attributed to the large particle size and lack of porosity for VT700. Figure 5b displays the reflectance spectra for both samples.

The optical absorption coefficient, α, of both samples were derived from the transmittance and reflectance spectra based on the following relationship^24,25^:

1 $$\alpha =\frac{1}{t}ln\left\{\frac{\left[{\left(1-R\right)}^{2}\right]}{T}\right\}$$

where *T* and *R* are the transmittance and reflectance, and *t* is the thickness of the film.

The optical band gap of sample VT400 and VT700 was determined using Tauc’s relationship between *α* and energy of incident photons exciting electrons from the valance band to the conduction band (*hν*)^24,26^:

2 $$\left(\alpha h\upsilon \right)=k{\left(h\upsilon -{E}_{g}\right)}^{n}$$

where k is an energy-dependent constant, ${E}_{g}$ is the optical band gap. The exponent *n* depends on the nature of transition responsible for the absorption, $n = 1/2,2,3/2\;or\;3$, which correspond to allowed direct, allowed indirect, forbidden direct or forbidden indirect transition. We initially tested all the possible type of transitions n-values by plotting ${\left(\alpha h\upsilon \right)}^\frac{1}{n}$ versus the incident photon energy $\left(h\upsilon \right)$. It was observed that $n = 1/2$ (direct allowed) transition displays the best slope fitting or tangent to the curve where the intercept occurred at $\alpha h\upsilon =0$. Figure 5c illustrates ${\left(\alpha h\upsilon \right)}^{2}$ versus $h\upsilon$ of samples VT400 and VT700 with direct allowed optical band gap values of 1.821 eV and 1.678 eV. The decrease in optical band gap with increasing substrate temperature is attributed to the increase in grain size as discussed above. These optical band gap values are consistent with those observed by Yu et al.^28^, where they synthesised high-quality VO_2_ thin films on silica substrates via radio frequency sputtering and plasma-enhanced chemical vapour deposition. They reported optical band gap values ranging from 1.54 to 1.74 eV. Similarly, Zhen-Fei et al.^29^ reported an optical band gap of 1.81 eV for thermochromic nanocrystalline VO_2_ thin films fabricated by magnetron sputtering and post-oxidation, which is in good agreement with that of VT400.

The imaginary refractive index or extinction coefficient (*k*) was also deduced from the absorption coefficient obtained from Eq. (1) and using the following relationship^29^:

3 $$k=\frac{\lambda }{4\pi }\alpha $$

Following this, the refractive index (*n*) of the films was determined from the reflectance (R) spectra by employing the following Eq.^27^:

4 $$ n = \frac{{\left( {1 + R} \right)}}{{\left( {1 - R} \right)}} + \left[ {\frac{{4R}}{{\left( {1 - R} \right)^{2} }} - k^{2} } \right]^{{1/2}}  $$

The real (*n*) and imaginary (*k*) refractive indices deduced from transmittance and reflectance spectra are shown in Fig. 5d. It is noted that real and imaginary refractive indices for sample VT700 are slightly higher than sample VT400. However, in both samples the complex refractive index decreases with increasing wavelengths from 250 to 2500 nm. These results are consistent with optical constants such as *n* and *k* obtained from VO_2_ thin films deposited on silica-soda-lime and silica-potash-soda using a UHV magnetron sputtering system, reported by Dai et al.^30^. Similarly, Kana et al.^31^ fabricated VO_2_ thin films onto various glass substrates by radiofrequency inverted cylindrical magnetron sputtering and then investigated temperature-dependent studies on optical constants. The refractive index and extinction coefficient measured at 30 °C ranges from 2.0 to 3.6 and 1.86 to 0.25 in the spectral range between 300 to 1600 nm^31^. These results suggest that optical constants of VO_2_ thin film depends on the fabrication conditions and techniques employed.

### MIR thermochromic properties and phase transition temperature control

**Figure 6:**
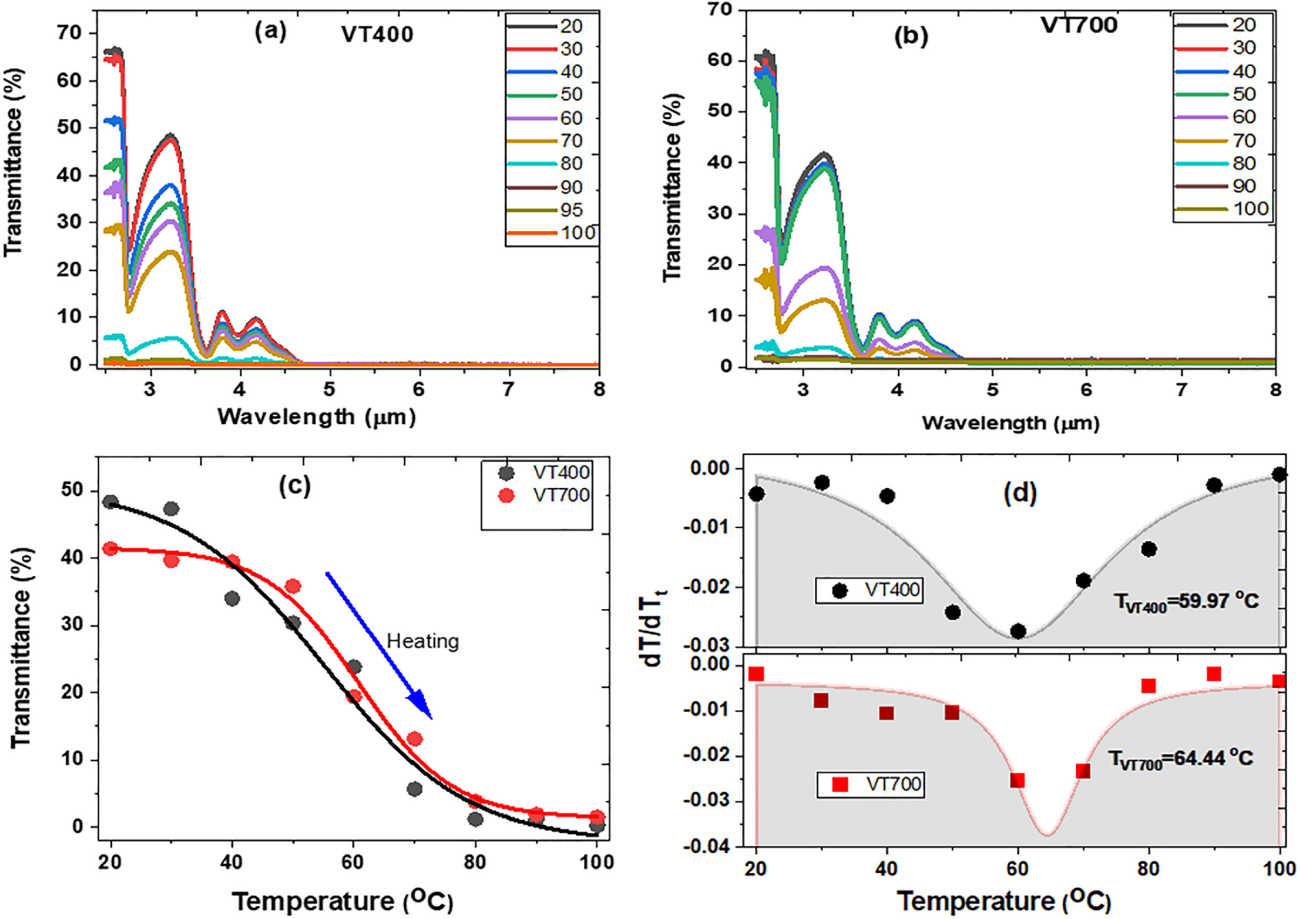
Temperature-dependent transmittance measurements (**a**) VT400 and (**b**) VT700. MIR transmittance as a function of sample temperature for samples VT400 and VT700. (**d**) The differential curves of transmittance to temperature verses heating temperature.

The MIR optical transmittance of the VO_2_ thin films as a function of temperature ranging from 20 to 100 °C were measured to evaluate their thermochromic properties and insulator-to-metal transition temperatures. Figure 6a,b show the transmittance behaviour in the 2.5 to 25.0 μm wavelength range obtained as a result of heating of the thin films. The thermochromic transition efficiency of the VO_2_ (M1) film is defined in terms of optical contrast factor, $\tau \left(\lambda \right)$, expressed as^32^:

$$\tau \left(\lambda \right)={\tau }_{LT}-{\tau }_{LH}$$

where ${\tau }_{LT}$, and ${\tau }_{LH}$ are transmittance at low and high temperatures, respectively, and λ is the MIR wavelength. For example, the optical contrast factors attained at transparency windows peaking at 2.6 μm and 3.2 μm are 66.26% and 48.15% for VT400, and 65.87% and 40.00% for VT700, respectively. According to Guinneto et al.^22^, the primary parameters affecting the contrast factor are particle size and morphology and the high contrast factor in the case of VT400 is attributed to the high porosity combined with the small grain size compared to sample VT700.

Figure 6c,d depicts transmittance obtained at 3.2 μm as a function of heating temperatures for both samples (VT400 and VT700). Sample VT700 reveals a sharp and abrupt switching hysteresis transmittance curve compared to sample VT400, which looks steeper in the transition region. This clearly shows that VT700 sample exhibits excellent MIR transmittance switching efficiency than the sample VT400. Moreover, the MIR transmittance is nearly reduced to zero below the transition temperature at 70 °C as illustrated in Fig. 6a,b. The differential curves of transmittance to temperature [i.e.$\{{dT}_{r}/dT\}$] is displayed in Fig. 6d, which was fitted with a Lorentz profile equation to ascertain metal-to-insulator transition parameters. The phase transition temperatures were determined to be ~ 60.0 °C and ~ 64.4 °C for VT400 and VT700 samples. Sample VT700 has a narrow hysteresis width of FWHM = 12.6 °C as related to FWHM = 33.7 °C of sample VT400. The phase transition temperatures are in good agreement with their heating temperature as a function of resistivity measurements as shown in Figure S2 (a) and (b (i) and (ii)) in the supplementary information. Consequently, such significant variation in transition temperature and hysteresis width between both samples fabricated at different substrate temperatures can be attributed to film discontinuity, density, porosity, crystallinity states, grain boundaries, defects, film particulates and thickness of the samples studied here^22,31^. For instance, the VO_2_ grain size increases with increasing the substrate temperature. This is due to the fact that particles are agglomerated at high substrate temperature to form a more compact thin film with minimising grain boundaries as illustrated in Fig. 2d from the TEM cross-section image. Notably, the transition temperature of VT700 is closer to that of the bulk VO_2_ (M) sample (68.0 °C). The thermochromic parameters attained from sample VT700 are identical to the results reported by Guinneton et al.^22^, who reported a thermochromic optical switching transition temperature of 68.0 °C and transition range of less than 10 °C for a VO_2_ film thickness of 120 nm. We however report a similar performance for a film of 5 times thicker than this grown using fs-PLD.

### MIR and LWIR emissivity

**Figure 7:**
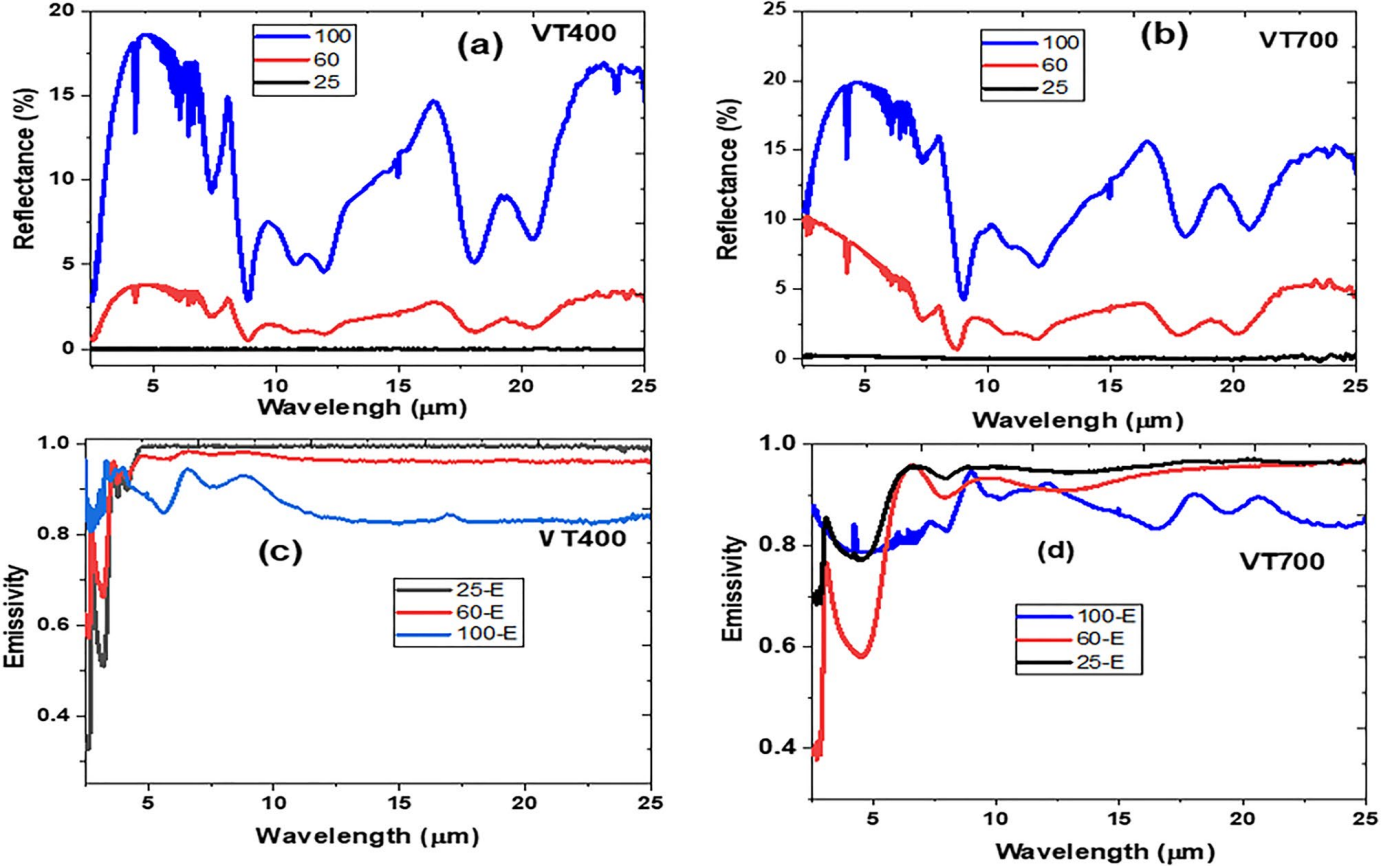
Variation in reflectance of the VO_2_ thin films at different temperature (**a**) VT400 and (**b**) VT700; Emissivity of VO_2_ thin film at different temperature (**c**) VT400 and (**d**) VT700.

Temperature dependent reflectance measurements at 25 °C, 60 °C, and 100 °C are illustrated in Fig. 7a,b. It can be seen that the VO_2_ thin films exhibit a significant rate of change of reflectance upon heating, which correlated with previous report by Guinneton et al.^15^. The transmittance and reflectance measured at temperatures of 25 °C, 60 °C, and 100 °C were used to determine temperature-dependent emissivity of the VO_2_ thin film. The emissivity as a function of wavelength was estimated by employing conservation of energies related to thermodynamic radiation characteristics expressed as^23^:

5 $$\varepsilon \left(\lambda \right)=1-\rho \left(\lambda \right)-\tau \left(\lambda \right)$$

where $\varepsilon \left({\varvec{\lambda}}\right)$, $\rho \left({\varvec{\lambda}}\right)$, and $\tau \left({\varvec{\lambda}}\right)$ represent absorptivity, emissivity, reflectance and transmittance.

According to Kirchhoff’s second law of thermodynamics, at equilibrium, the emissivity of a material must be equal to the absorptivity, $\boldsymbol{\alpha }$**,** at constant wavelength (${\varvec{\lambda}}$**)** , and temperature (T).

6 $$\varepsilon \left(\lambda \right)=\alpha \left(\lambda \right)$$

Figure 7c,d shows the infrared emissivity of VT400 and VT700 films at different temperatures revealing thermochromic properties. It is noted that the emissivity is highest at lower temperatures and decreases at a higher temperature for sample VT400 as compared with sample VT700. Such difference in emissivity values are attributed to variance in optical contrast, reflectivity and transmittance. Thus, the rougher thin film surface has a lower reflectivity and higher scattering owing to more grain boundaries or higher porosity.

The initial feasibility studies suggest that variable thermo-optical emissivity properties can be achieved passively within a small change in temperature in the MIR from VO_2_ (M1) thin film prepared using fs-PLD. It is important to mention that the change in emissivity of VO_2_ (M1) thin film decreased as temperature increases. These results correlate with Gomez-Heredia et al.^33^, who synthesised VO_2_ thin films onto sapphire and silicon substrates using a pulsed laser deposition technique with a KrF pulsed excimer laser. The authors demonstrated decreasing in emissivity as a function of increasing temperature in the MIR wavelength. Moreover, it’s been suggested that the MIR emissivity properties of VO_2_ (M1) thin film depends mainly on the infrared optical properties of the substrate. For example, Benkahoul et al.^34^. synthesised VO_2_ thin films on various substrates including quartz, silicon, and polished minor-like Al employing RF reactive sputtering of vanadium target. The authors reported that temperature dependence of the emissivity of VO_2_ thin film deposited onto highly IR reflective Al substrate is opposite to samples deposited on quartz and silicon substrates. This is attributed to increase in reflectance with temperature for VO_2_ thin film deposited onto quartz substrate as compared to VO_2_ film on Al substrate, which decreases with increasing temperature.

## Conclusion

A fs-PLD technique enables realising scalable manufacturing of thicker VO_2_ (M1) thin film within a shorter timescale from less expensive V_2_O_5_ target material as compared with the conventional methods. This technique was employed to deposit VO_2_ (M1) thin films onto silica substrates at different substrate temperatures. Surface morphology studies using SEM imaging reveals that sample fabricated at a substrate temperature of 400 °C (VT400) comprises small nanoparticles or grain sizes of about 12 nm. Conversely, as the substrate temperature increased to 700 °C (VT700), the particles agglomerated to form a film of larger particle size with an average value greater than 360 nm. The TEM and XRD characterisations confirmed that VO_2_ thin films deposited on silica substrate consist of polycrystalline and single crystal systems, respectively, with a monoclinic orientation of (011). The increase in substrate temperature (sample VT700) leads to an increase in particle or grain size with reduced grain boundaries and film thickness, minimum porosity defects on the surface and cross-section. Subsequently, the optical absorption edge decreases with an increase in substrate temperature due to lack of porosity defects on the thin film surface and cross-section. This leads to decrease in optical band gap and slight increases in refractive index from the visible to NIR spectrum. Furthermore, sample VT700 exhibits high-quality thermochromic properties and best insulator-to metal transition temperature switch of 64.4 °C and hysteresis width of 12.6 °C at 3.2 μm wavelength. On the other hand, sample VT400 shows a better modulation of emissivity under heating from 25 to 100 °C. Consequently, these results confirm the tunable optical and thermochromic properties of these VO_2_ thin films on silica substrate fabricated by fs-PLD technique with significant potential for developing smart window applications.

## Supplementary Information

Supplementary Information.

## Data Availability

All experimental deposition conditions and characterization procedures, methods and data are provided in the text and supplementary information. Any clarifications will be available by contacting the corresponding author.
