# Supplementary material for: Infrared optical properties modulation of VO2 thin film fabricated by ultrafast pulsed laser deposition for thermochromic smart window applications
Source: Sci Rep. 2022 Jul 6;12:11421. doi: 10.1038/s41598-022-15439-5 (PMC9259692; doi:10.1038/s41598-022-15439-5)
Supplement: Supplementary file 1 — Supplementary Information. [file 41598_2022_15439_MOESM1_ESM.docx]

**Infrared Optical Properties Modulation of VO_2_ Thin Film Fabricated by Ultrafast Pulsed Laser Deposition for Thermochromic Smart window Applications**

**Eric Kumi Barimah^1^, Artitsupa Boontan^1^, David P. Steenson^2^, Gin Jose^1^**

**^1^**School of Chemical and Process Engineering, University of Leeds, Clarendon Road, Leeds LS2 9JT, U.K.

**^2^**School of Electronic and Electrical Engineering, University of Leeds, Clarendon Road, Leeds, LS2 9JT, U.K.

**Supporting Information**

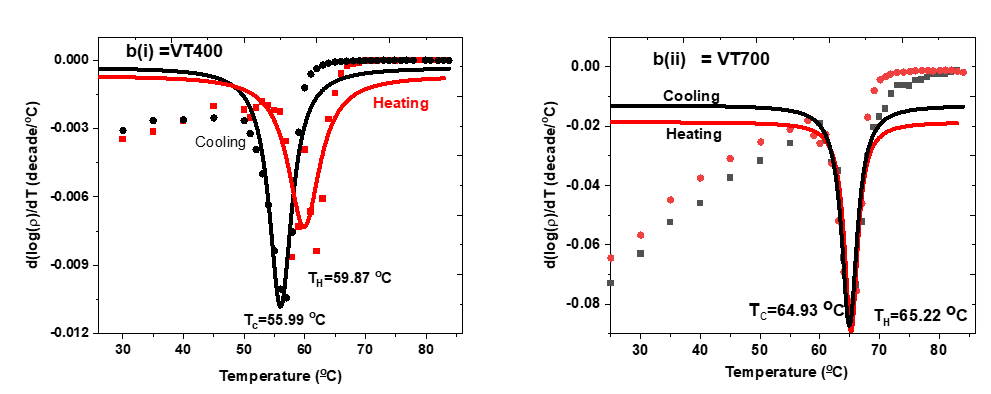


Figure S2: (a) The electrical resistivity (ρ) as a function of the temperature of samples VT400 and VT700. (b) (i) and (ii) First derivative of log_10_ (ρ) versus temperature of samples VT400 and VT700 fitted with Lorentz equation. The red and black curves indicate the heating and cooling of the VO_2_ thin films deposited on the silica substrate.

Table 1: The metal-to-insulating temperature of as-deposited VO2 thin films on silica substrate

| Sample ID | Heating transition  Temperature (T_H_ ^o^C) | Heating transition  Temperature (T_C_ ^o^C) | Average transition  Temperature (T_ave_ ^o^C) |
| --- | --- | --- | --- |
| VT400 | 59.87 | 55.99 | 57.93 |
| VT700 | 65.22 | 64.93 | 65.08 |
